# A dental dilemma: bear teeth reveal a spatial gradient in age of primiparity, but temporal trends muddled when bears grew fast

**DOI:** 10.1098/rsos.252109

**Published:** 2026-10-07

**Authors:** Andrew N. Tri, David L. Garshelis, Pamela L. Coy, Carolin A. Humpal, Karen V. Noyce

**Affiliations:** Forest Wildlife Populations and Research Group, Minnesota Department of Natural Resources, Grand Rapids, MN, USA

**Keywords:** American black bear, age of first reproduction, cementum annuli, food production, growth rates, methodological limits, primiparity, reproduction, teeth, *Ursus americanus*

## Abstract

Age of primiparity (AOP) in American black bears (*Ursus americanus*) is driven by nutrition. We estimated AOP from patterns of cementum annuli in female bear teeth (more than 16 000), which were collected from hunters in Minnesota, USA, over five decades. AOP was delayed in northern Minnesota and hastened southward, matching the increasing food availability. In the core of the bear range, AOP remained stable at 5.3 years, whereas along the range edge, AOP increased from 4.5 years in the 1970s to 5.0 years in the 2010s. We posed five hypotheses to explain this enigmatic trend; (i) change in tooth readers, (ii) declining foods, (iii) increased density-dependent effects, (iv) hunters killing fast-growing juveniles, and (v) limits to the technique used. We rejected the first four hypotheses by comparing tooth readers and exploiting data on bear foods, age-specific bear masses, population trends and observed AOPs of radio-collared bears in each region. We concluded that food increased along the range edge, spurring faster-growing bears, which had harder to interpret cementum annuli. We previously developed and tested this technique on slower-growing bears. Teeth from harvested bears are a valuable source of reproductive information, but the technique may fail when employed beyond the tested limits of the bears' nutrition.

## 1. Introduction

Reproduction is closely linked to nutrition in both domestic and wild animals [1–3]. Better nutrition speeds growth and maturation and reduces the age of primiparity (AOP). In long-lived species, where AOP may vary by multiple years, even a slight change in average AOP can have substantial effects on population growth [4,5].

The AOP of American black bears (*Ursus americanus*; hereafter, black bears) varies widely across their range in North America but generally declines from west to east, complementing a west-to-east increase in litter size and body size [6,7]. AOP varies with individual nutritional status, and at the population level, mean AOP reflects the habitats and foods available [8–10]. Black bears in eastern North America tend to begin reproducing at 3–5 years of age; cub production at 3 years (mating at 2 years) is common [11–13] and possible at 2 years [11] where hard mast is abundant (e.g. oak acorns (*Quercus* spp.), beechnuts (*Fagus* spp.) and hickory nuts (*Carya* spp.)), or anthropogenic food sources are available [13–15]. In the western USA and Canada, first reproduction at 5–6 years is more typical [7,16,17]. AOP of black bears is more spatially variable and sensitive to nutrition than litter size or litter frequency [18–20].

A standard method for assessing black bear reproduction is to visit the dens of radio-collared females during winter. Black bears give birth to cubs while hibernating, generally in January. Researchers locate the dens of radio-collared bears, chemically immobilize the mother and count, sex and weigh the cubs. Cubs remain with their mother for approximately 17 months; cubs that survive their first year den with their mother for a second winter [18]. Thus, by visiting dens each year, researchers can directly observe and document reproduction and cub survival, as well as the body conditions of both mother and cubs.

This technique is widely used with black bears, which can be safely handled in dens, but it can be problematic in southern states where bears do not hibernate soundly and may abandon dens when approached by researchers [21–24]. Moreover, it requires substantial effort every year to visit winter dens and maintain a continuous sample of radio-collared bears, particularly in populations where research animals are killed by legal hunters each year. AOP is particularly difficult to measure and monitor at the population level using radio-collared bears because it only occurs once in each animal's lifetime and known-age radio-collared juveniles are often killed before reaching primiparity.

In Minnesota, USA, we conducted a long-term study of black bear reproduction that entailed visiting 20–80 dens each year for more than four decades (1981–2025). We obtained data from study sites spread across the geographic range, representing very different habitats; however, the amount of data varied greatly by site and year, related to the number of radio-collared bears.

Here, we sought to examine broad-scale spatial and temporal trends in AOP using a technique that provides vastly more data with less effort. In the early 1990s, we discovered that when female black bears in Minnesota nursed cubs, it altered the spacing of annual lines (annuli) in their teeth [25]. Teeth that are sectioned into thin slices and stained show a series of dark lines within the cementum of the root, each corresponding to altered cementum deposition during hibernation. Between each dark line is a lighter-staining band representing the growth of cementum during the active season. Based on a sample of radio-collared bears with known reproductive histories, we observed that the light band of cementum is approximately half the width in years when females are nursing cubs than in years when they are not [25]. Black bears generally produce cubs at 2-year intervals, and although cubs remain with their mother for 1.5 years, they nurse only during their first year; hence, the width of the light cementum bands alternately narrows and widens every other year, creating the appearance of pairs of dark annuli with wider light bands on each side (figure 1). We found that the reproductive histories of radio-collared bears in Minnesota could be accurately reconstructed by examining the spacing pattern of annuli in their teeth. Moreover, interpretations of annuli in black bear teeth from Maine and Yosemite National Park, California, also closely corresponded to the known reproductive histories of individual bears [25]. Similarly, another researcher measured the width of cementum bands in the teeth of radio-collared black bears with known reproductive histories at three study sites in Arizona and concluded that during years when mothers were nursing cubs, these bands were consistently less than 50% as wide as in years between the birth of litters or when a full litter died [26]. Researchers examining teeth of Asiatic black bears (*Ursus thibetanus*) and polar bears (*Ursus maritimus*) demonstrated that this technique could have applications for understanding reproduction in other bear species as well [27–29]; however, this technique is unreliable for assessing brown bear (*Ursus arctos*) reproduction [30].

**Figure 1.**
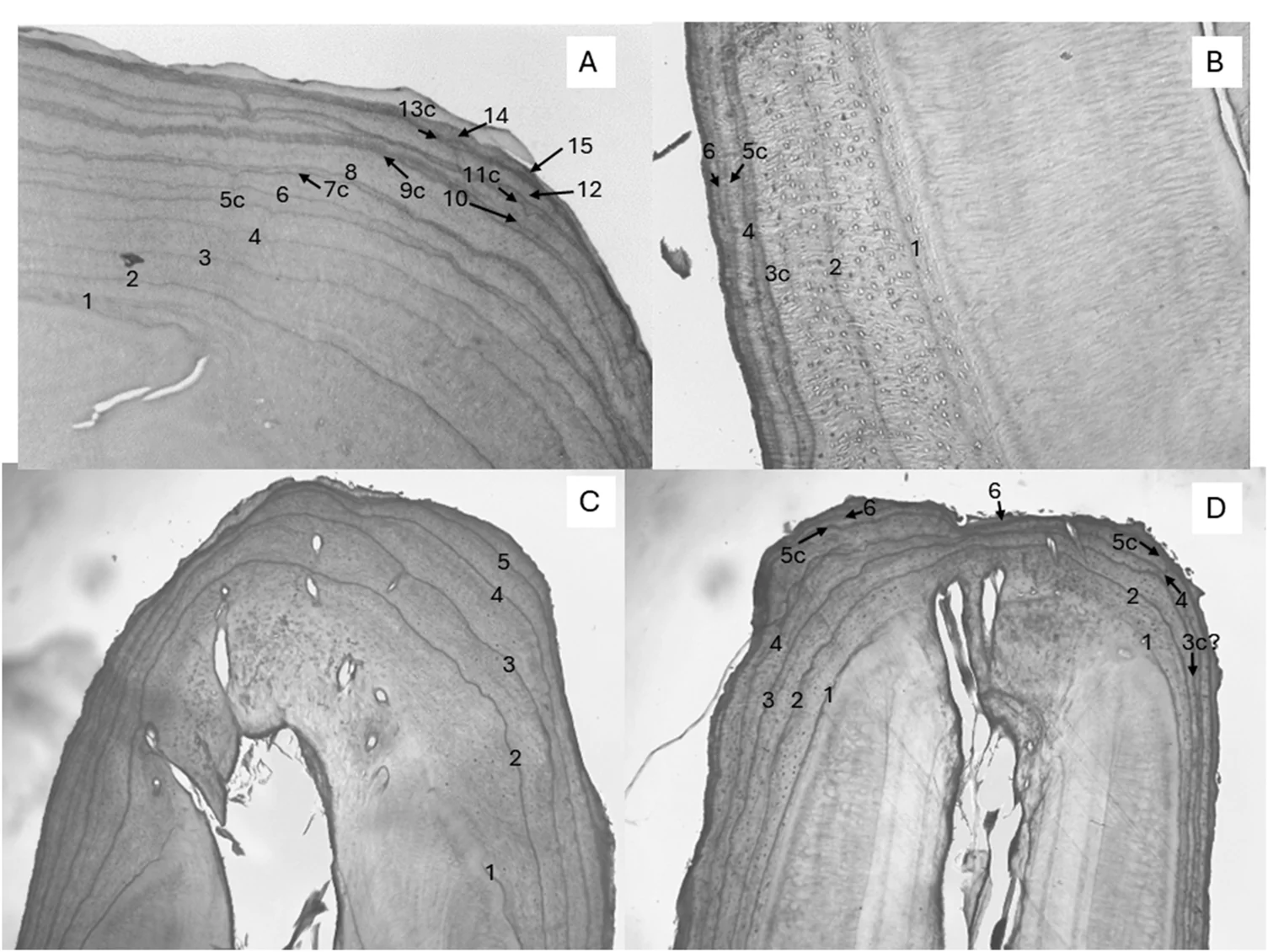
Sectioned and stained roots of teeth, showing annuli used to estimate age and age of primiparity (AOP) in female black bears, Minnesota, USA. Ages are labelled, and cub years (interpreted from annular spacing) are denoted with ‘c’ after the age. All slides were observed at 40× magnification unless otherwise noted. Panel (A): tooth of a 15-year-old bear with AOP at 5 and additional cub litters at 7, 9, 11 and 13 years; this tooth was collected in March, so the new dark annulus is at the edge of the tooth. Panel (B): tooth of a 6-year-old bear with AOP at 3 and an additional litter at 5 (100× magnification); this tooth was pulled in June while a light cementum layer was being deposited. Panel (C): tooth of a 5-year-old bear with no indication of reproduction in the cementum, killed in September. Panel (D): tooth of a 6-year-old bear with an uncertain age of first reproduction at 3 or 5 years, killed in September. The right-hand side shows a possible first litter at 3 with a definite litter at 5, and the left-hand side shows a definite litter at 5 but no obvious litter at 3.

One complication in reconstructing reproductive histories from bear teeth, however, is that whereas cementum bands narrow markedly in years of nursing cubs, they also narrow as a bear gets older and in particularly poor food years [25,26,31–34]. This can create errors, especially if one is trying to count every litter [35]. Here, however, our only objective was to identify the first litter. When our research team first applied this method in Minnesota in the early 1990s, using a sample of more than 1500 teeth from harvested female bears greater than or equal to 4 years of age, we observed a definitive AOP in 76% of the sample, no reproduction (by the time of the bear's death) in 11%, and the pattern was not clear in 13% [20,25]. Since then, we have continued measuring AOP from teeth obtained during each year's hunter harvest, aiming to measure long-term trends.

We have amassed thousands of additional teeth from the hunted bears. Here, we examined this long-term dataset for spatial and temporal patterns in AOP. In some respects, our new findings simply corroborate the already well-known relationship between food availability and AOP. However, in examining the data further, we uncovered an enigmatic finding; the mean AOP on the edge of the Minnesota bear range—where natural and human-related foods tend to be most abundant—increased through time, suggesting that foods in this area were declining, even though there was no indication of this occurring. We were thus uncertain whether the teeth highlighted an environmental change that was otherwise undetected or if the method based on the teeth had misled us. Here, we present multiple streams of data to evaluate five simultaneous working hypotheses [36,37] to explain this counter-intuitive finding.

## 2. Methods

### 2.1. Study area

This study encompassed the entirety of the black bear range in Minnesota (figure 2). The core bear range occurs in the north-eastern two-fifths of the state (approx. 98 000 km^2^) and consists of large expanses of forest with little agriculture. The far northern forest is dominated by aspen (*Populous tremuloides* and *P. grandidentata*), balsam fir (*Abies balsamea*), pine (*Pinus banksiana*, *P. strobus* and *P. resinosa*), paper birch (*Betula papyrifera*) and red maple (*Acer rubrum*) trees. Moving towards the edge, this transitions to a patchy mix of aspen and mixed northern hardwood forests, some places with an oak component (*Q. rubra, Q. macrocarpa and Q. ellipsoidalis)*. The secondary bear range, characterized by lower bear density and more fragmented habitat, occurs along the southern and western edges of the primary range (approx. 21 500 km^2^). Typical cover is small woodlots intermixed with agriculture (less than 30% forested). Northern hardwoods, oak forests and oak savannas are the predominant forests, although on the northwestern fringe of the range, aspen parklands with oaks are common on ancient beach ridges, and lowlands of deciduous shrubs and conifers are extensive. Feed corn, sweet corn, hay, oats and soybeans comprise approximately 48% of the landscape in secondary bear range [38–40].

**Figure 2.**
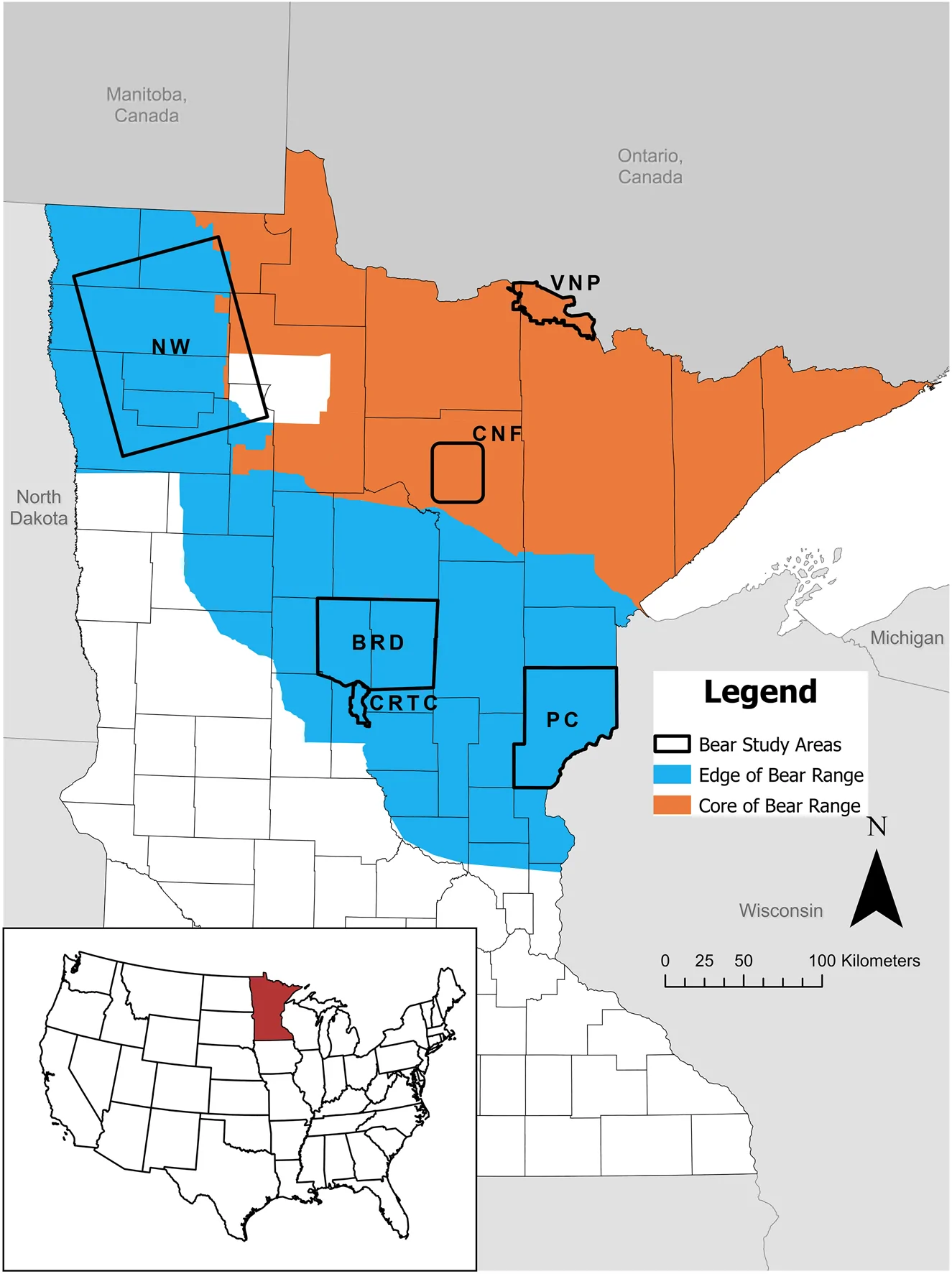
Core and edge of Minnesota bear range where teeth were collected by hunters and the six study sites where we studied reproduction of radio-collared bears (1981–2025). Core range: Chippewa National Forest (CNF) and Voyageurs National Park (VNP). Edge of range: Camp Ripley Training Center (CRTC), Brainerd (BRD), Pine County (PC) and Northwest (NW).

Important summer bear foods throughout Minnesota are fleshy fruits (wild sarsaparilla (*Aralia nudicaulis*), raspberry (*Rubus strigosus*), blueberry (*Vaccinium* spp.), blackberry (*Rubus* spp.), juneberry (*Amelanchier* spp.) and cherries (*Prunus* spp.)). Principal autumn foods include beaked and American hazel (*Coryllus cornuta* and *C. americana*), dogwood (*Cornus* spp.), and, where available, oak acorns [41,42]. The abundance and diversity of summer and autumn foods are lowest in the northern and north-eastern parts of the range and increase dramatically to the south and west [43,44].

### 2.2. Bear hunting

Since the modern bear hunting seasons started in 1971, bears in Minnesota have been legally hunted during autumn (September–October). Hunters must purchase a license. Females accompanied by cubs can be legally harvested, but not cubs born that year. More than 90% of hunters attract bears using bait [43]. Hunters must register their bears and extract a premolar for age estimation.

The Minnesota Department of Natural Resources (MDNR) managed the harvest by dividing the bear range into Bear Management Units (BMUs), the number and boundaries of which varied over time. Most BMUs had annually adjusted quotas on the number of hunting licenses, although along the edge of bear range, two BMUs (the northwesternmost and southeasternmost BMUs) had no restrictions on the number of hunters. Hereafter, we refer to the five BMUs along the edge of the bear range as the edge and the other eight BMUs as the core of the bear range.

### 2.3. Estimating age of first reproduction from teeth

From 1975 to 2023, bear hunters in Minnesota submitted a tooth (generally the first upper premolar) from their harvested bears to the MDNR. These were used to estimate the bear's age and thereby generate a harvest age structure for use in modelling the population trajectory. In most cases, hunters submitted teeth via mail in a special envelope, where they indicated the sex of the bear and the date and location of the kill. In return, they were provided with the age of their bear and, since 1980, an embroidered patch to incentivize compliance. Compliance was also enhanced through a series of steps; (i) making it mandatory, (ii) providing reminder letters, and (iii) assessing penalties for those failing to submit a tooth in multiple years.

Each tooth was decalcified in 10% hydrochloric acid and sectioned into longitudinal slices (12–15 µm) using a freezing microtome or a cryostat. We mounted the sections on albumin-coated slides (1980s–early 2000s) or VWR Superfrost Plus slides (late 2000s–2023; Avantor, Radnor, PA) and stained them using Harris hematoxylin. We estimated age-at-harvest by examining multiple sections of each tooth under a compound microscope at 40× and 100× magnification and counting the dark annuli in the cementum of the root, from the dentine–cementum interface outward. Biologists have used this process to age bears for decades [45–48].

Permanent first premolars begin to come in during a black bear cub's first summer (they lack deciduous precursors) and are fully erupted by September [34,48], which corresponds with the beginning of denning in Minnesota; therefore, the first dark cementum annulus often occurs very close to or along the dentine–cementum interface. Because the root tip is not closed in September, it can sometimes be difficult to see the first annulus on the dentine–cementum interface. For bears killed during September–October, interpreting the outermost cementum layer also requires extra care, as it can appear as either a light cementum band representing the current active season or as a newly forming dark annulus if cementum accretion had already slowed during pre-hibernation.

We estimated AOP by examining the spacing of the first 3–9 annuli, looking for the first noticeably thin band of light cementum (resulting in a close pair of dark annuli; figure 1). We examined multiple places around the root in multiple sections of the same tooth using the same method developed in the 1990s on Minnesota bears [25]. We note that this change in cementum width only becomes evident if the mother nurses her cubs through weaning, which occurs in early autumn; thus, we define AOP as the age at which bears produce their first litter in which at least one cub survives at least until autumn.

The first few bands of cementum are always wide, thereafter narrowing with age; hence, tooth readers must distinguish thin bands representing cub-rearing from the progressive thinning of bands with age (figure 1A,B). We assigned an AOP only if a band was at least 50% thinner than the previous one and was followed by a significantly wider band. To make this comparison, the bear had to live at least 1.5 years past its AOP (figure 1, [20,25]). Hence, we did not attempt to detect AOP that occurred in the year of death (cubs born that January and mother hunted in September).

Even in bears killed two or more years after first giving birth, AOP was sometimes difficult to discern. Figure 1D shows a tooth with different pairing of annuli on different sides of the tooth, yielding an AOP at either 3 or 5 years of age. In such cases of uncertainty, we used a random number generator using a uniform distribution to assign AOP as one of the two possibilities (less than 1% of all cases).

### 2.4. Calculating mean age of primiparity

If all bears lived long enough to give birth to their first cubs, a simple mean of their AOPs would provide an accurate estimate of the reproductive capacity of the population. However, in a hunted population, many bears die before giving birth (as is evident from their teeth (figure 1C)). A simple mean calculated from bears that lived to primiparity would therefore be biased low because those that would have reproduced at an older age had a higher chance of being killed before giving birth relative to those reproducing at younger ages.

We calculated mean AOP using an unbiased estimator developed by Garshelis *et al.* [49], which considers not only bears that reached primiparity but also those of potential reproductive age that died before giving birth (nulliparous). This method is based on calculating the percentage of nulliparous females of each age that produced their first cubs at that age. Nulliparous bears were counted up to a year before their age of death because, as noted above, the relative width of the last light cementum band was hard to assess for bears killed in autumn. For example, bears that died at 5 years of age and did not have cubs by the age of 4 years (figure 1C) were used to calculate the proportion of 4-year-olds and also the proportion of 3-year-olds, giving birth. The youngest known AOP in Minnesota (i.e. radio-collared bear of known age) was 3 years old, so to detect this in the teeth, a bear had to be at least 4 years old; consequently, all bears shot before age 4 were excluded from our analysis. Any teeth that showed no indication of reproduction by age 8 were censored at age 8, as our radio-collared data indicated that it is rare for a bear in Minnesota to have its first cubs at 9 or 10 years of age [49].

This estimator was developed to eliminate the bias that would be caused by bears dying in the years just prior to their first reproduction (in our case, ages 3–8). However, it also eliminates the bias that could be caused by a changing age structure in the living population, which is bound to occur in a population subject to varying hunting pressure. The estimator relies only on the percentage of nulliparous bears of each age that produced cubs, treating each age as a separate sample; it is not affected by how large that sample is or how many from one age class died before reaching the next.

Each record in the dataset was coded for the bear's sample number, year and age at death (integer years; thus, 4 years 9 months = 4 years), year of birth, if the bear was nulliparous at harvest, AOP (if detected), and the decade in which primiparity occurred. We coded each record to BMU and whether that BMU was in the core or edge of the bear range. We assumed that the BMU where the bear was shot represented the general area where it lived and reached primiparity.

We used a non-parametric product limit estimator [50] within the ‘survival’ package [51] in R 4.5.1 [52] to compare AOP (and associated standard errors) among decades and regions. While originally designed to estimate survival or time to failure, we adapted it for use here because, like death, having a first litter of cubs occurs only once in a bear's lifetime. We note that whereas these estimators have previously been applied by monitoring individuals through time (e.g. radio-collared bears followed until death or removal from the sample), the teeth data are analogous in that the annuli patterns captured a complete record of an individual's reproduction until its death.

For all analyses, we grouped the data by the decade in which the bear's AOP occured or did not occur. Each year in the reproductive history leading up to the AOP (or death prior to AOP) could have occurred in the same decade or overlapped two decades. Moreover, these events could have occurred well in the past, depending on the age of the bear when it was killed. For example, a bear shot at 28 years of age in 2022 was born in 1994; if it was nulliparous at ages 3, 4 and 5, that would have been during the 1990s, whereas AOP at age 6 would have been in the 2000s.

### 2.5. Mass of harvested bears

Hunters optionally weighed the dressed carcass (organs removed) of their harvested bear and reported it on the envelope in which they submitted the bear's tooth, enabling us to match masses with age. We used the masses of female bears to assess trends in bear nutrition, spatially and temporally.

During the 1980s, when the MDNR provided stations with scales where bears could be weighed, approximately half of the submitted tooth samples had an associated weight in pounds. In the decades since then, hunters could optionally weigh their harvested bears on their own, and about a third provided a weight with the tooth. We recognize that this sample may have been biased, particularly towards heavier bears; however, we assumed that the bias did not change appreciably over time. Also, although hunters were instructed to provide a weight only if the bear was actually weighed, we were concerned that some reported weights were estimates. To alleviate this possible issue, we excluded reported weights that ended in 50 or 00. We converted all weights (to mass (kg)). We used notched boxplots with 95% confidence intervals of the medians to compare, by decade, age-specific masses of juveniles (ages 1–3) and reproductive adults (ages equal to or greater than 6) by decade in the core and the edge of the range.

### 2.6. Reproductive data from radio-collared bears

We used data from radio-collared bears to compare with reproductive data gleaned from hunter-submitted teeth. We collected reproductive data on radio-collared female black bears continuously since 1981. We trapped bears in summer and fitted them with either a VHF or GPS collar. We anesthetized all collared bears in their dens each winter and recorded whether they had cubs (born in the den) or yearlings (1-year-olds denning with their mother). We put radio collars on female yearlings in dens to monitor their future reproduction.

We studied radio-collared bears at six sites in different parts of the Minnesota bear range (figure 2), representing a diversity of conditions in terms of hunting pressure, habitat, land ownership and natural food production [18]. Two study sites were in the core of the bear range; Chippewa National Forest (central bear range, sampled 1981–2025) and Voyageurs National Park (northern bear range, sampled 1997–2022). Four study sites occurred along the edge of Minnesota's bear range; Camp Ripley Training Center (sampled 1991–2025), Pine County (1991–1993), Brainerd (2022–2025) and Northwest (2007–2021). Voyageurs National Park and Camp Ripley Training Center were unhunted, although bears often left these areas in autumn and were vulnerable to hunting. The four study sites along the edge had more oaks, and all but Camp Ripley had more agriculture (including crops that bears consume, mainly corn, oats and sunflowers) than the two core study sites.

### 2.7. Hypothesis testing

As we show below, the reproductive dataset from examination of the hunter-submitted teeth confirmed that AOP broadly reflected the availability of food on a landscape level. It also indicated an apparent increase in AOP through time for bears living along the edge of the bear range, but not for those in the core. This trend was unexpected, given the general abundance of bear foods on the edge of the range. We posed five hypotheses for this apparent trend: (i) a change in how teeth were read (i.e. changing AOP was not real, but an artefact of who was reading teeth), (ii) changes in food conditions, (iii) changes in bear density, (iv) hunter selection for and removal of the fastest growing juveniles, and (v) inherent limitations of the method. Below, we explain these hypotheses and how we tested them.

#### 2.7.1. Hypothesis 1: differences between tooth readers

Two authors read the tooth samples, each during a different period of years (Reader 1 (PLC): 1975–2006 and Reader 2 (CAH): 2007–2023); therefore, we considered the possibility that temporal trends observed in AOP (on the edge) resulted from differences in how these two readers interpreted patterns in the annuli. Thus, this hypothesis predicted that Reader 2 tended to estimate AOP as older than Reader 1, but only for bears on the edge. Reader 1 pioneered the method of estimating AOP from bear teeth using samples of bears with known reproduction [25] and was also part of the field team studying radio-collared bears, so was familiar with nutritional influences on bear reproduction and spatial differences in food availability across Minnesota. Reader 1 trained Reader 2 in interpreting AOP from annuli.

We tested for differences between tooth readers by comparing their readings of age-at-harvest and AOP from the same set of teeth. Reader 2 examined a set of 82 archived slides that were originally read by Reader 1 in 2004. Reader 1 examined a set of 79 archived slides that were originally read by Reader 2 in 2018 and 2019. At the time of the test, both readers had 15 years of experience interpreting AOP from bear teeth, but Reader 1 had more (26 years total) experience in aging bear teeth. We used the estimates of age-at-harvest and AOP of Reader 1 as the standard against which we compared the estimates of Reader 2. We looked for patterns in disparities between readers using pairwise permutation tests for both the edge and the core.

#### 2.7.2. Hypothesis 2: reduced foods

We hypothesized that AOP increased along the edge of the range owing to a long-term decline in food abundance in this specific area. We assessed natural food abundance across the bear range using an annual survey (1984–2023) conducted by personnel (*n* = 20–80 each year) from the MDNR, US Fish and Wildlife Service, US Forest Service, tribal biologists, county foresters and other individuals who worked in the field and were familiar with fruit-bearing plants in their work areas [53]. We asked participants to numerically (0–4) rate the abundance of 14 bear food-producing species or species-groups in their work area each year, and (separately) the fruit production of each species that year [41,42]. We multiplied these two ratings to obtain a fruit abundance score for each species. We derived a composite summer food index by summing the fruit abundance scores of wild sarsaparilla, juneberries, cherries, blueberries and raspberries—five summer-ripening species that, on average and through time, were the most abundant (and most used by bears [41]). We calculated the mean summer food index in the core and the edge of the bear range each year and then fitted linear regression models in R 4.4.1 [52] to detect change through time. We similarly looked for changes through time in acorn abundance (oak abundance × productivity) and in the composite oak + hazel + dogwood productivity index—key autumn foods that have a disproportionate effect on reproduction [44,53].

Row crops (primarily corn; *Zea mays*) are also important autumn foods for bears along the edge of the range but not the core [54]; therefore, we considered the possibility that this food was the primary driver in the observed increase in AOP along the edge. We downloaded the annual county-wide estimates of corn yield (metric kt) and area planted in corn (km^2^; not including silage corn, which represents less than 5% of corn grown for livestock in Minnesota and is typically harvested in mid-summer, so it is not available to bears for much of their hyperphagia period) for all the counties along the edge of bear range from the US Department of Agriculture National Agriculture Statistics Service [55]. We fitted linear regression models in R 4.4.1 [52].

As a second test of this hypothesis, we compared median mass (and 95% confidence intervals), by decade, of harvested female bears (as provided by hunters). If increased AOP along the edge of the range was owing to a reduction in foods, age-specific masses of bears should have declined along the edge (but not the core).

#### 2.7.3. Hypothesis 3: density-dependent effects

Increased bear density could affect AOP by increasing competition for food and reducing average food intake per bear, thus slowing growth and maturation rates [56]. To test whether bear numbers increased at the edge, but not the core, we estimated population trend using Downing population reconstruction, a method based on the ages of harvested bears [57]. This method provides a robust reflection of population trends, even with modest violations of assumptions [58]. Also, similar to hypothesis 2, this hypothesis predicted that age-specific mass of harvested bears would decline over time along the edge but not the core.

#### 2.7.4. Hypothesis 4: hunter selection for larger juveniles

AOP may have increased owing to hunters selectively removing juvenile bears that would have had a young AOP. Bear hunters in Minnesota often have a choice of which bear they shoot because several bears may visit their bait, and they tend to select larger individuals. Thus, if food availability increased, juveniles would grow faster, and hunters would be apt to shoot the largest of these, which would have been the ones to mature at a younger age. If hunters kill a high proportion of these fast-growing juveniles, this could result in the counter-intuitive situation where better nutrition results in disproportionately few fast-growing bears living to maturity, and hence an older average AOP. This was most likely to occur on the edge, where food was already more plentiful. This hypothesis predicted that, over the four decades, hunter-harvested juvenile bears on the edge increased in mass, but the mass of adult bears (i.e. bears not harvested as juveniles) would not have increased at the same rate, and may have declined, if the slower-growing bears never caught up on their growth curve owing to their slower start, smaller stature, where they lived, or behaviour with respect to use of anthropogenic food sources [59]. In turn, lighter-weight adults raise smaller cubs which mature later [19].

#### 2.7.5. Hypothesis 5: methodological limitations

Our method of detecting AOP relied on discerning whether a band of light cementum was much narrower than bands on either side (figure 1). However, a confounding issue is that irrespective of the effects of nursing cubs, cementum bands are wide when the bear is young, and then progressively narrow (figure 1C), and this natural narrowing coincides with years when AOP normally occurs; moreover, the width of the bands in young bears relates to how fast the bear is growing structurally. Here, we posited that in particularly fast-growing juvenile bears, readers might be less apt to detect AOP because the narrowing is less distinct. Thus, if nutrition was increasing along the edge and bears were growing especially fast, readers might miss the real AOP and misidentify the second litter as the first. Accordingly, this hypothesis predicts that along the edge, the real AOP was declining owing to better nutrition, whereas we erroneously perceived it to be increasing.

We did not have a sample of teeth to directly test this. However, we had known AOP from radio-collared bears near the edge and in the core of the range, which could be compared with the AOP discerned from the teeth of hunter-killed bears. If we were missing early AOP as per this hypothesis, then the proportion of radio-collared bears along the edge with an AOP of 3 years should be significantly higher than that proportion detected in teeth; however, in the core, the radio-collared and tooth-based estimates of AOP should match.

## 3. Results

### 3.1. Spatial variation in age of primiparity

From 1975 to 2023, hunters submitted 16 468 teeth from 4+ year-old female black bears, from which we estimated age-at-harvest and AOP. We ascertained that 11 465 had had cubs at least a year before they were killed and 5003 had not (table 1). Our sample included bears born as early as 1955, but teeth showing litters born in the 1950s and 1960s were too few (*n* = 86) to yield reliable decadal estimates of mean AOP; therefore, we limited results to the 1970s–2010s.

**Table 1.** Calculation of average AOP, based on interpretation of teeth collected from hunter-killed black bears, 1975–2023. Calculations include all bears that reached reproductive age, whether or not they had cubs by the time they were killed^*^.

age	no. nulliparous females available to reproduce	no. producing	no. shot before reaching next age	% nulliparous females producing (A)	% females in population available to produce (B)	% females in population producing their first cubs (C)	age weighted by proportion reproducing (D)	cumulative proportion reproduced by given age
3	16 468	773	2479	4.7%	100.0%	4.7%	0.1	4.7%
4	13 216	3673	1678	27.8%	95.3%	26.5%	1.1	31.2%
5	7865	4110	611	52.3%	68.8%	36.0%	1.8	67.1%
6	3144	1922	184	61.1%	32.9%	20.1%	1.2	87.2%
7	1038	750	51	72.2%	12.8%	9.2%	0.6	96.5%
8	237	237	0	100%	3.5%	3.5%	0.3	100.0%
sum		11 465	5003			100%		
mean							5.13	

^*^ Number of 3-year-old nulliparous females available to produce is the total number of teeth examined from bears older than or equal to 4 years of age at the time of death. Subtracting the number that produced cubs at 3 and the number that died at 3 without producing cubs yields the number of nulliparous females available at 4, and so on. Column (A) is calculated from no. producing/no. nulliparous females available to produce. The percentage of the population available to produce (B) is 100% at 3 year of age and then diminishes by the percentage that produced in the age cohort prior. Column C is calculated by multiplying columns (A) and (B). Column (D) is calculated by multiplying the age by column (C), and summing these yields the mean AOP (had bears lived to reach AOP). We assumed that any nulliparous bears shot at age 7 would have had cubs at 8. We censored bears in which we could not detect reproduction by age 8.

Statewide, and across the five decades, 82.5% of female bears had their first litter at 4–6 years of age, 12.8% at 7 or 8 and 4.7% at 3; overall mean AOP was 5.13 years (table 1). Geographically, the percentage of females that produced cubs by 5 years of age increased from northeast to southwest; 40–55% (variation by BMU and decade) in the far north, 55–75% through a central band and greater than 75% in the south-western-most edge BMUs (figure 3).

**Figure 3.**
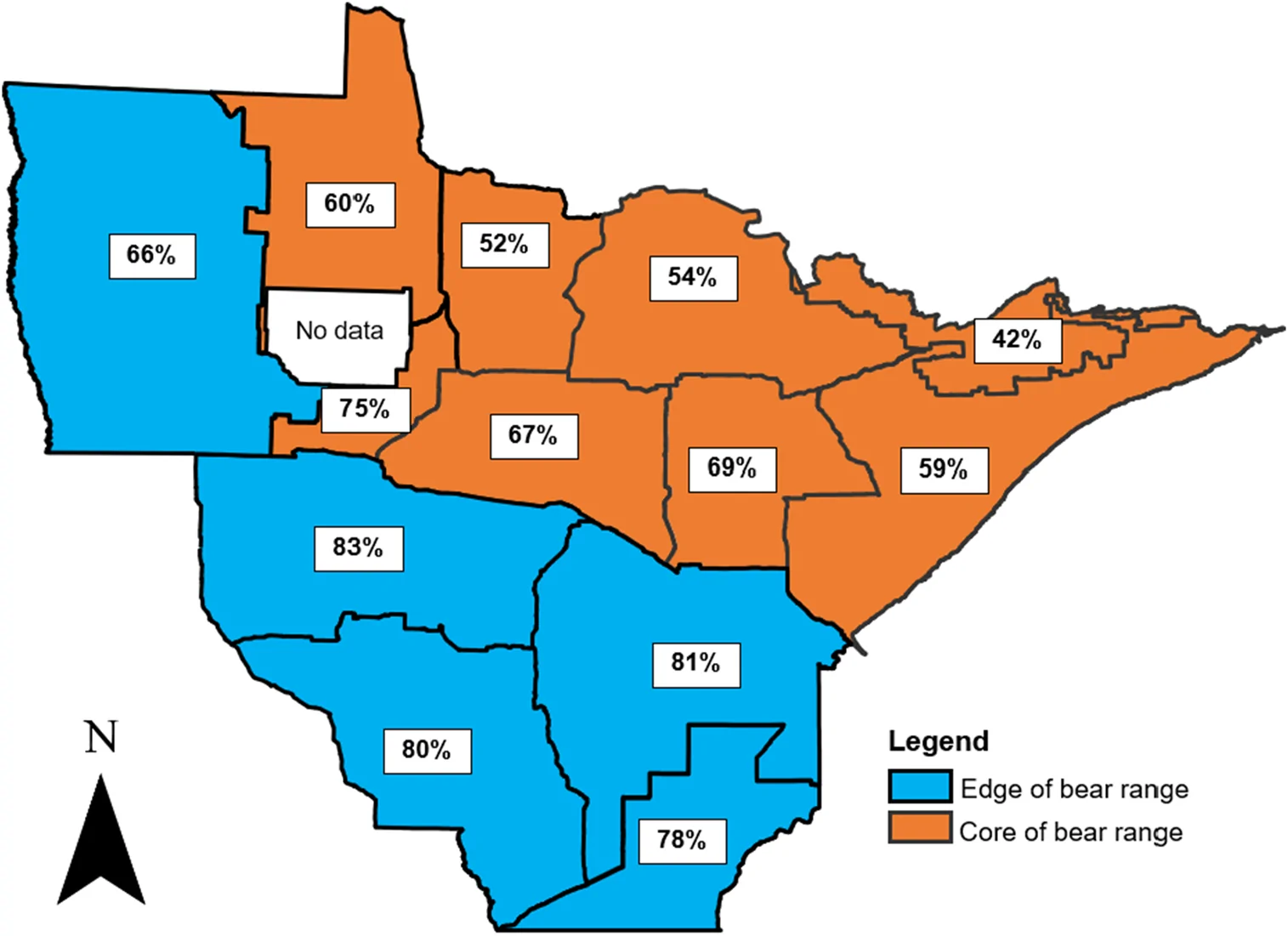
Overall mean percentage of black bears that reproduced by age 5 in each BMU in Minnesota, based on the evaluation of annuli in more than 16 000 teeth submitted by hunters, 1975–2023.

### 3.2. Spatiotemporal variation in age of primiparity

Across decades, average AOP was stable in the core bear range (5.33 years ± 0.03 s.e.) other than a small decline in the 2010s (5.16 ± 0.04 s.e.). Along the edge, AOP was consistently younger, and increased from 4.47 (± 0.10 s.e.) years in the 1970s, to 4.66 (± 0.05 s.e.) in the 1980s and 1990s, and 5.00 (± 0.09 s.e.) in the 2010s (figure 4). Accordingly, the percentage of bears that reproduced by the age of 5 was mainly stable in the core (44–48% through the 2000s, increasing to 58% in the 2010s), but declined on the edge (from 88% in the 1970s to 74% in the 2010s; figure 4). On a finer scale, all BMUs in the core range had stable (or in one case declining) AOP over this time, whereas AOP in all edge BMUs increased by 0.3–0.8 years. We examined five hypotheses to explain this unexpected trend in the most food-rich part of the bear range.

**Figure 4.**
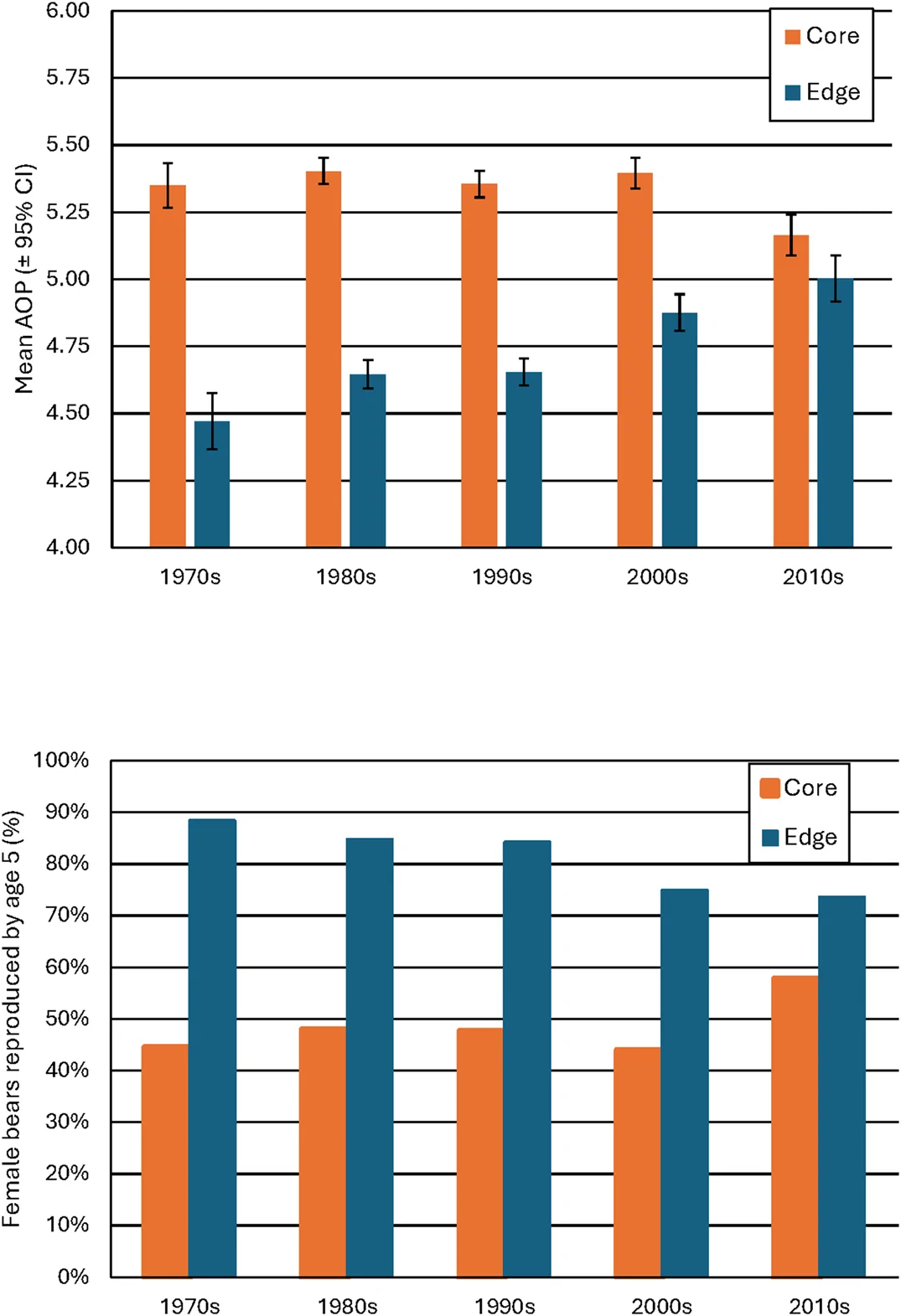
Temporal variation in mean age of primiparity (± 95% confidence intervals; AOP, top panel) and the percentage of female black bears that reproduced by age 5 (bottom panel), derived from examination of hunter-harvested teeth backdated to their decade of first reproduction, in the core versus edge of bear range in Minnesota.

#### 3.2.1. Hypothesis 1: difference between tooth readers

We compared AOP estimates between the two tooth readers for 75 teeth from bears killed in the core and 86 teeth from bears killed along the edge in 2004, 2018 and 2019 (table 2). AOP for this sample of teeth, as determined by both readers, ranged from 3–7 years for the core and 3–6 for the edge. Reader 2′s AOP estimates exactly matched Reader 1 for 88% of teeth in the core and 91% on the edge (90% areas combined). We found no statistical difference between AOP estimates of readers for teeth from the core (*Z* = 0.12, *p* = 0.261) or the edge (*Z* = -0.06, *p* = 0.341). Of those differences that did arise, Reader 2′s estimates were not consistently older or younger than those of Reader 1 (table 2).

**Table 2.** Comparison of AOP discerned by two readers from the same sample of Minnesota bear teeth, submitted by hunters in 2004, 2018 and 2019. Reader 1 developed the technique, which was used as the baseline for comparison.

		Reader 2 AOP compared with Reader 1		
area	Reader 1 AOP (years)	younger	same	older
core	3	0	4	0
	4	0	14	3
	5	0	38	0
	6	3	7	0
	7	3	3	0
	sum	6	66	3
	% of total	8%	88%	4%
edge	3	0	15	2
	4	1	36	2
	5	1	26	1
	6	1	1	0
	sum	3	78	5
	% of total	3%	91%	6%

In the process of comparing readers, we discovered that they used slightly different criteria for discerning AOP. Reader 1 looked for the first light cementum band that was half or less than the width of the previous band, followed by a wider band. Reader 2 focused more on the apparent pairing of dark annuli but was not as stringent as Reader 1 about the width of the intervening light band. Reader 1 also used the pairing of dark bands as a clue, but with the caveat that it occurred consistently around the tooth rather than just in one spot.

We also discovered that the two readers differed in some of their age-at-death estimates, specifically in cases where the last dark annulus was very close to the edge when the bear was killed in autumn. Although the readers did not try to interpret AOP from the last annulus, it appeared that cub production in the year of death led to differences in how they counted the last dark annulus, leading to a discrepancy of 1 year in their age estimation for a portion of the sample. This discrepancy could have affected the age assigned to masses of 4- and 5-year-old bears (some of which produced cubs), so we excluded these. However, for mass comparisons across time, we grouped all bears older than or equal to 6 years to form one large adult category, where the 1-year age reading discrepancy for some bears would have a negligible effect.

#### 3.2.2. Hypothesis 2: reduced natural foods

We observed no decline through time in the composite summer food index in either the core or the edge of the bear range (figure 5, top panels). Conversely, summer foods seem to have increased over the past decades, and markedly so in the core, where availability of summer berries has consistently been higher than on the edge. There was no statistically significant change in autumn food productivity (dogwood, hazel and oak) in either the core or edge (figure 5, middle panels), although it was consistently higher on the edge. Acorn abundance was much higher on the edge, but with no statistically significant trend through time, whereas in the core, it increased statistically through time (*p* < 0.001; *r*^2^ = 0.47), driven by increasing abundance of mature oak trees (figure 5, bottom panels). Conversely, corn production (metric kt) on the edge increased by 400% since the 1980s, and the land area planted with corn (ha) increased by 250% (figure 6). There is more corn within bear range now than ever before, nearly all on the edge.

**Figure 5.**
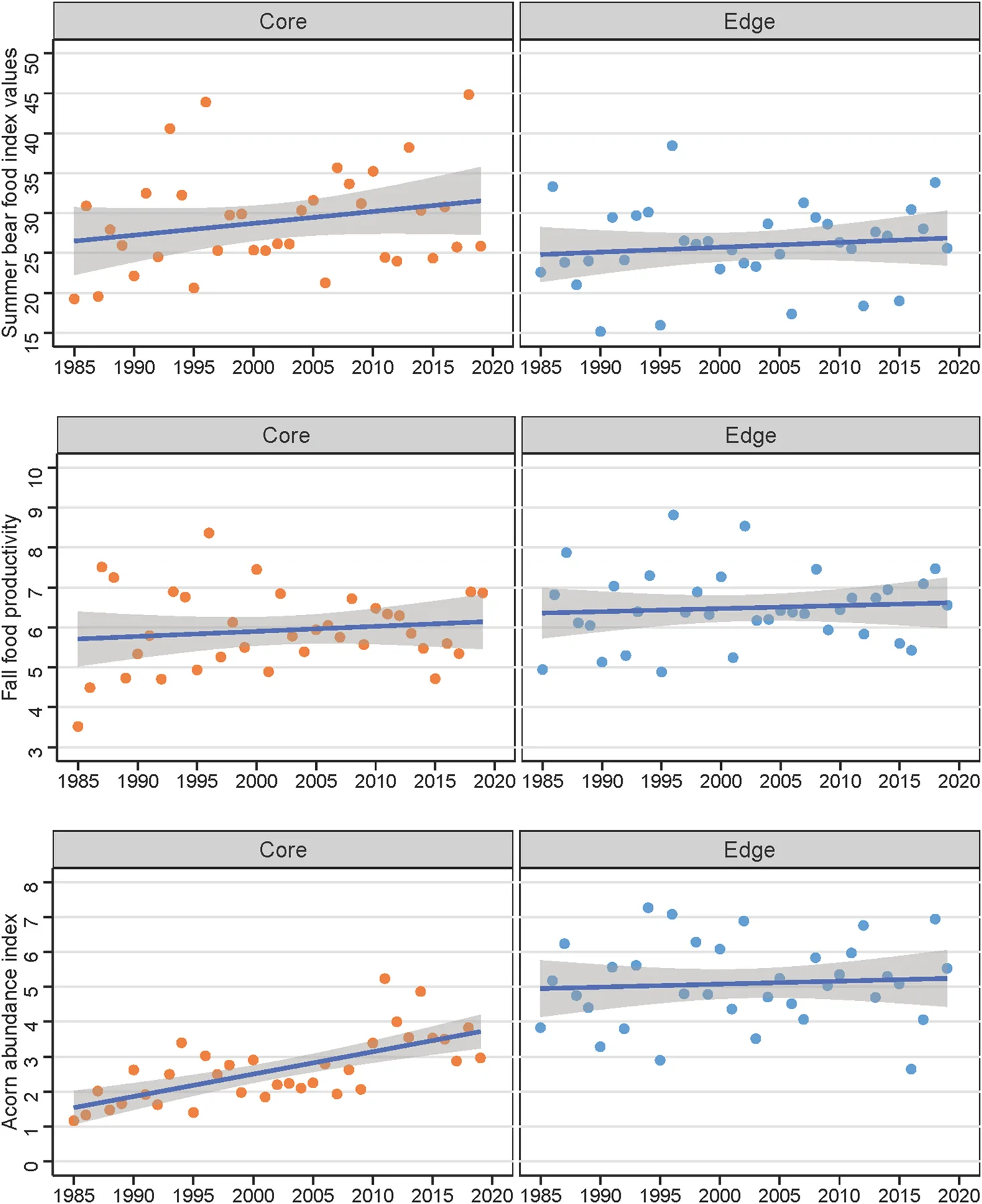
Variation in natural foods of black bears, 1985–2020, in the core versus the edge of bear range in Minnesota, as rated by field personnel (*n* = 20–80) in annual surveys. The top panels show the yearly mean summer food index (summed scores of species abundance × productivity) of wild sarsaparilla, chokecherry, juneberry, blueberry and raspberry). The middle panels show mean autumn food productivity (sum of dogwood, hazel and oak productivity scores). The bottom panels show the mean acorn abundance index (oak abundance × productivity).

**Figure 6.**
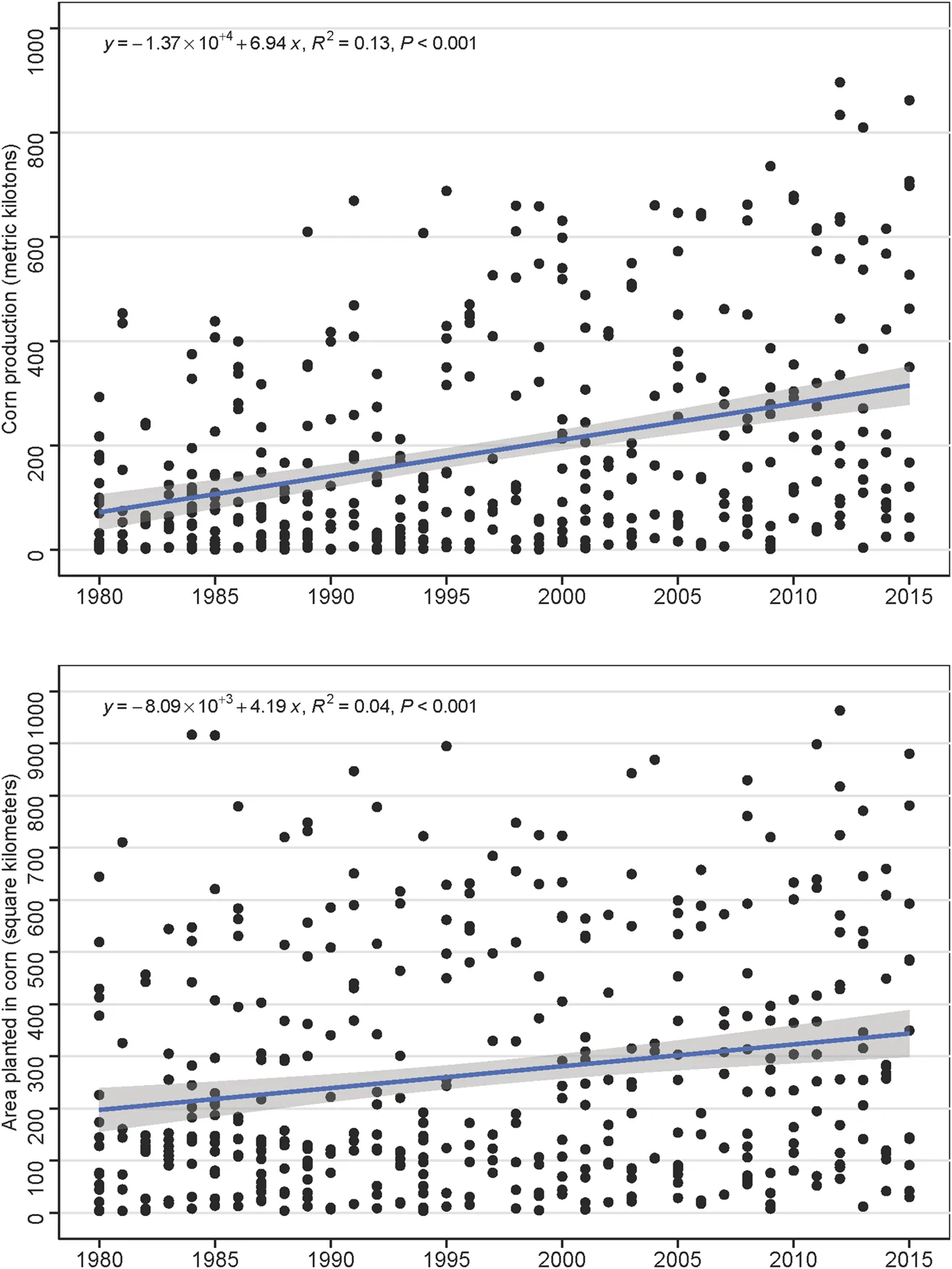
Corn production (metric kt) (top panel) and area planted (bottom panel) on the edge of bear range in Minnesota (1980–2015). Each data point represents an estimate of annual corn production for a county in the dataset. Both trend lines indicate a noisy but significant increase in corn on the landscape. Corn was not widely grown as a commercial crop in the core range.

For each age group (1, 2, 3 and 6+), female bears on the edge were heavier than those in the core, and this difference grew through time. Age-specific masses increased each decade from the 1980s to the 2010s in both the core and the edge, but this increase was greater on the edge (figure 7). The median dressed mass of 3-year-old females on the edge increased by approximately 20 kg from the 1980s to 2010s; by the 2000s, they weighed nearly as much as 6+ year-old bears in the core during the 1980s (figure 7).

**Figure 7.**
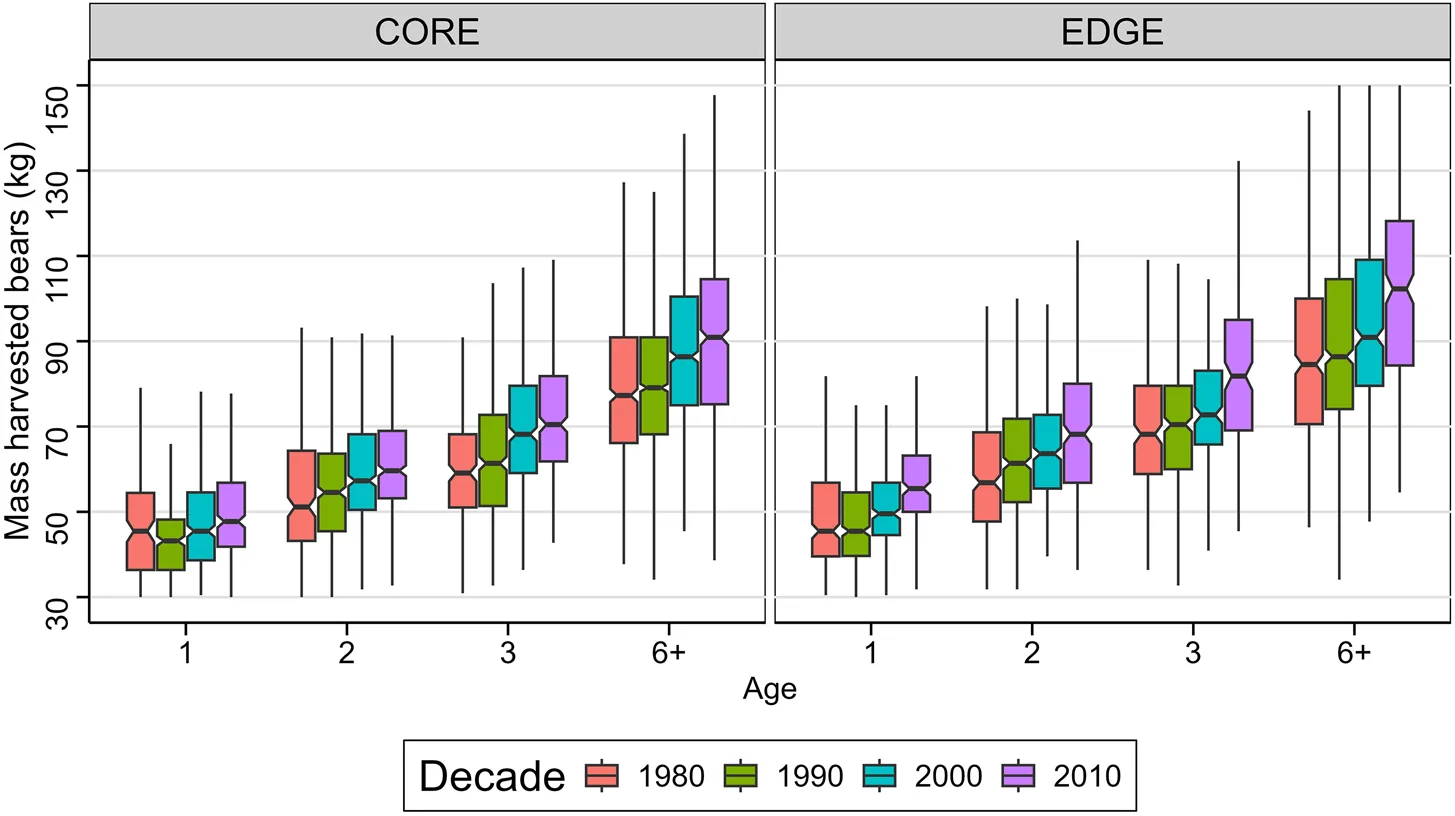
Dressed mass (carcass without entrails) of female bears reported by hunters when they submitted their bear tooth, by decade, in the core versus edge of Minnesota's bear range. We matched hunter-reported mass with age read from the tooth. The notch represents the 95% confidence interval of the median, the boxes represent the interquartile range (25% and 75% quartiles) of the median, and the whiskers represent the range of the data. Hunters reported this information voluntarily, but sample sizes were substantial in each decade (1980s *n* = 2437, 1990s *n* = 4391, 2000s *n* = 3144, 2010s: *n* = 2280).

#### 3.2.3. Hypothesis 3: density-dependent effects

The statewide bear population doubled from the 1980s to the late 1990s, then declined precipitously, nearly halving over 15 years owing to high harvest. After about 2013, the population steadily recovered. The core and the edge showed similar trajectories (figure 8). These trajectories were unrelated to either the stable or increasing trends in AOP evidenced in the core and the edge of the bear range, respectively, or to any trends in the mass of hunter-harvested bears (figure 7).

**Figure 8.**
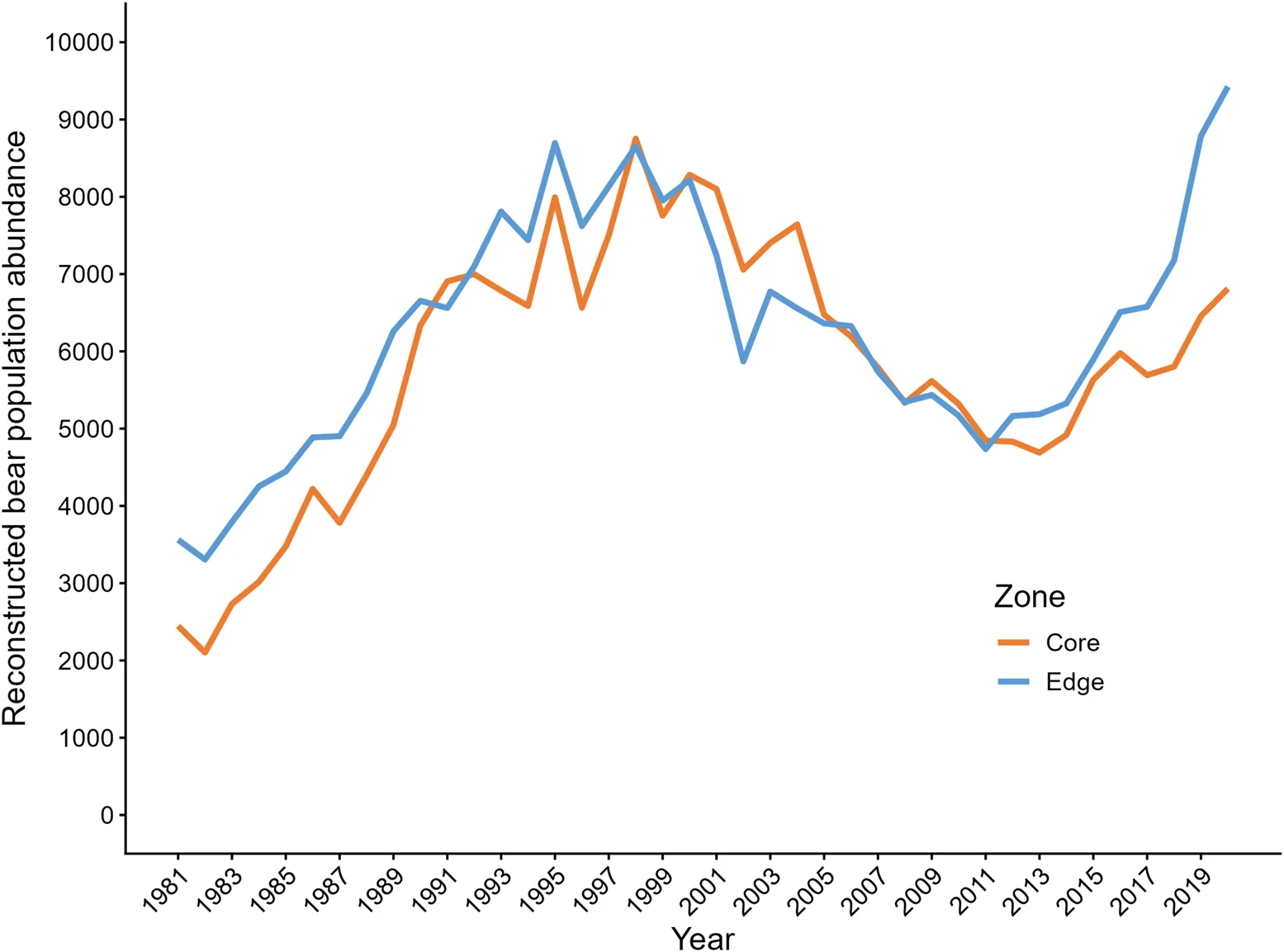
Trends in abundance of black bears in the core and the edge of Minnesota's bear range, based on population reconstruction, 1981–2020.

#### 3.2.4. Hypothesis 4: hunter selection for larger juveniles

The percentage of the female harvest composed of 1–3-year-olds trended upward from the 1980s to the mid-2010s, for both the core and the edge, but was always higher on the edge (48–73% core versus 69–85% edge; figure 9). This could have been indicative of a changing age structure in the living population, and/or hunters being more prone to shooting these younger bears because they were larger in later decades (figure 7). Even if hunters were selectively removing the largest ones, this could not have explained an increase in AOP (delayed maturity) on the edge because the surviving bears, which reached maturity, were heaviest in the 2010s (6+ year-olds, figure 7).

**Figure 9.**
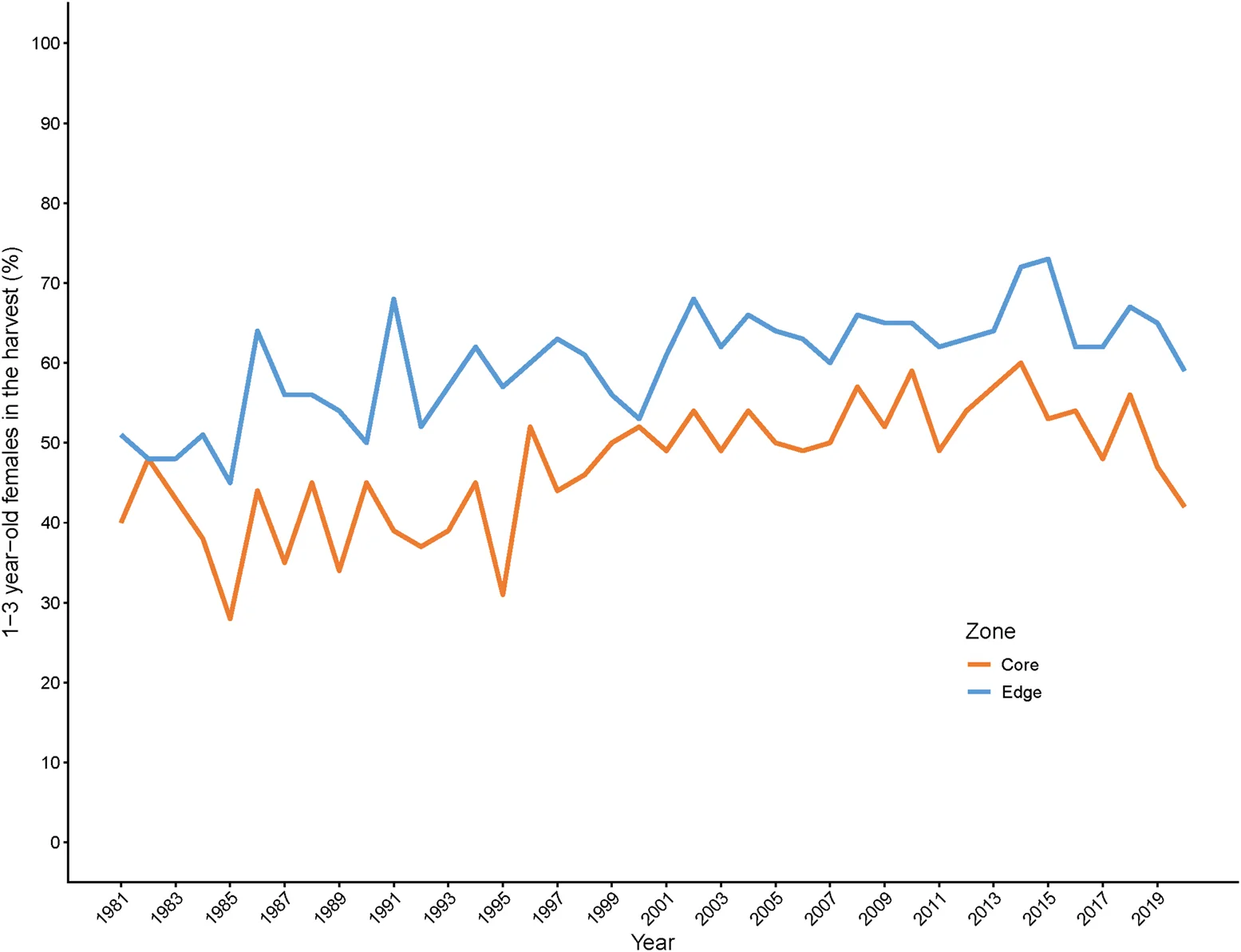
Percentage of female bear harvest composed of juveniles (1–3 years old) in the core versus the edge of Minnesota's bear range, 1981–2020. Data were obtained from teeth submitted by hunters.

#### 3.2.5. Hypothesis 5: methodological limits

In the core of the bear range, AOP data from radio-collared bears and the teeth of hunter-harvested bears were consistent, indicating that very few females (3%) produced surviving litters at 3 years of age and less than 30% had surviving litters at 4 (figure 10). Conversely, at the edge, a much higher proportion of radio-collared bears produced surviving litters at 3 years of age (48%) than gleaned from the teeth (8%). The difference between collared bears and teeth was less extreme for AOP at 4 years of age, but still indicative that, on the edge, we may have missed 7% of first litters at this age by examining lines in teeth (figure 10).

**Figure 10.**
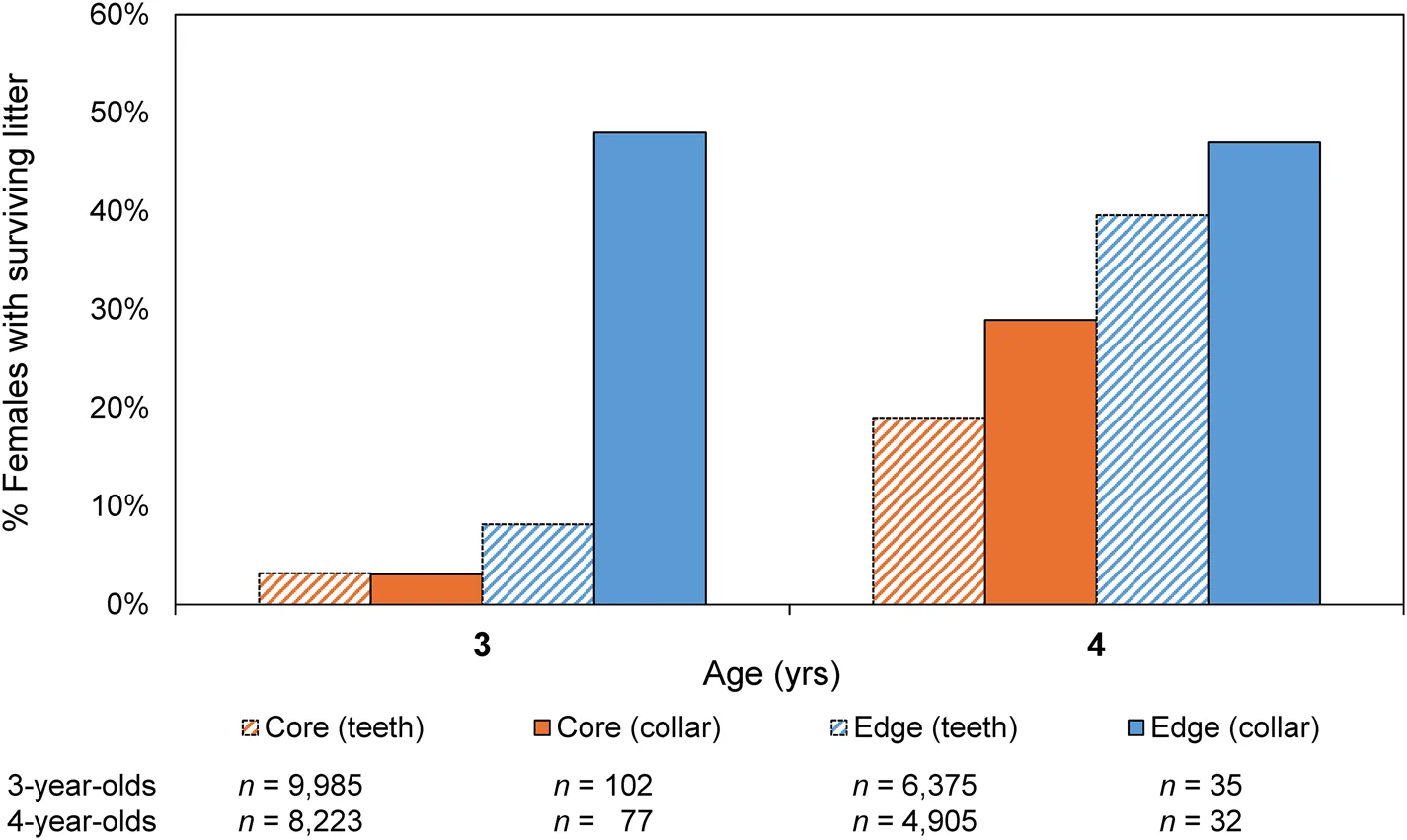
Percentage of 3- and 4-year-old female black bears that produced a surviving first litter, based on observations of radio-collared bears versus data gleaned by examining teeth from hunter-killed bears in the core and the edge of Minnesota's bear range. The sample sizes reported (*n*) refer to the number of bears available to reproduce at a given age.

## 4. Discussion

### 4.1. Attributes of using teeth to discern age of primiparity

The teeth of hunter-killed bears, which are routinely collected by management agencies across North America, provide a trove of data that can signal shifting age structures and reproduction on a large scale, requiring far less time and effort than any field studies ever could. Moreover, each year's sample provides additional data from the past (e.g. AOP of a bear killed at 25 years of age occurred two decades before). Although age becomes harder to read in teeth of older bears owing to the progressive thinning of the outermost cementum bands, the spacing of annuli in the early years of life is preserved; therefore, deciphering AOP is not affected by the age at death.

By contrast, assessing AOP of collared bears in dens involves weeks of effort in the field each year over many years, only to obtain far smaller and less spatially representative samples than the statewide tooth collection. We are unaware of any long-term field studies of bears that have registered significant trends in AOP through time, in part owing to sample size limitations. A shift in AOP of just 0.25 years in a decade would be significant in terms of population growth, especially in hunted populations where many bears are harvested before reaching AOP. However, detecting such a small change in a field-based study would be nearly impossible.

### 4.2. Hypotheses explaining spatiotemporal variation in age of primiparity

In the 1990s, we learned that AOP could be discerned from the pattern of cementum annuli of bears [25], and by applying this method to teeth of hunter-harvested bears, we learned that AOP varied spatially in Minnesota, consistent with availability of bear foods [20]. Specifically, lower AOP (earlier maturation) of bears living in the southern and western parts of the range relates to greater abundance of fall foods (primarily acorns). Two decades of additional data beyond our earlier work confirmed the same spatial variation in AOP, strengthening our confidence in the technique. However, we were surprised at the apparent increase in AOP through time along the edge of the bear range, where both natural fall foods and anthropogenic foods are more abundant than in the core. An increase of 0.5 years in the mean AOP in edge bears, if real, might necessitate a reduction in harvest pressure in this region. In addition, our monitoring of bear foods and our field monitoring of radio-collared bears failed to alert us to this decrease in reproductive potential. We spoke to wildlife managers and foresters with decades of field experience in the region about this apparent trend, and none could provide a rational explanation. Anecdotally, they portrayed the area as still having an abundant and diverse array of bear foods (especially oaks and corn that are rich in calories and influence bear body growth and reproduction [60–62]). This prompted us to examine a suite of plausible hypotheses, three related to the natural system and two related to our methodology.

We acknowledge that our tests of these hypotheses were done *post hoc*, using mainly data that we had previously collected or data available to us through other sources. We rejected hypotheses with predictions that were inconsistent with observations from these multiple streams of independent data (table 3). The only experiment we conducted was the comparison of tooth readers. Although we had no true controls, the situation itself provided a sort of control in that the temporal increase in AOP occurred only along the edge, whereas the core remained stable. Therefore, we rejected hypotheses that could not explain this spatiotemporal difference.

**Table 3.** Hypotheses to explain increasing AOP from the 1970s to the 2010s along the edge but not the core of black bear range in Minnesota, as discerned from spacing of cementum annuli in bear teeth submitted by hunters.

no.	hypothesis	predictions	observations	support for hypothesis?
1.	change in tooth readers midway through the study led to different interpretation of AOP, but only in the edge of the bear range.	Reader 2 (2007–2023) assigned AOP at older ages than Reader 1 (1975–2006), specifically for bears on the edge, which tended to reproduce at earlier ages than the core.	no statistical difference between readers for AOP. Reader 2 rarely assigned older AOP than Reader 1 on the edge.	no
2.	decline in foods on edge of the bear range caused slower growth in females and delayed their AOP.	abundance of bear foods declined on the edge of the bear range but not in the core.	bear foods did not decline. Corn production significantly increased on the edge and oak production increased in the core.	no, opposite trend
		female masses declined through time along the edge but not in the core.	masses increased in both the core and the edge, but more along the edge.	no, opposite trend
3.	density-dependent effects depressed growth and delayed AOP along the edge.	bear population increased on the edge but not in the core.	over four decades, the bear population doubled, declined by half, and increased again in both edge and core.	no
		female masses declined through time along the edge but not in the core.	masses increased in both the core and the edge, but more along the edge.	no, opposite trend
4.	hunters on the edge selectively killed large juveniles, and as these became more common, fewer early maturing bears survived to adulthood.	masses of juvenile females increased through time, but on the edge, masses of adult bears remained stable or declined.	masses of juveniles increased, but so did masses of reproductive adults, both on the edge and the core.	no
5.	increased foods led to faster growth and caused wider spacing of the first few annuli, making AOP at 3 years of age harder to detect. More bears reproduce at 3 on the edge than the core, so this error led to a misperception of increasing AOP on the edge.	masses of juvenile females on the edge increased through time, and were heavier than core bears.	masses of juvenile females on the edge increased steadily through time, ultimately equalling the mass of many adult core bears.	yes
		proportion of AOP at 3 detected from teeth was lower than that observed directly in radio-collared bears. The disparity between the radio-collar data and tooth data was greater on the edge than the core.	on the edge, 48% of radio-collared bears produced cubs at 3, whereas from the tooth samples we detected only 8% (suggesting we missed 83% of 3-year-old reproduction using teeth). In the core, 3% had cubs at 3, and collar and tooth data were similar.	yes

#### 4.2.1. Hypothesis 1: difference between tooth readers

Inferring AOP from the spacing of cementum annuli involves a degree of subjectivity; therefore, we expected some discrepancies between our two tooth readers. However, the readers mainly agreed (90%), and where differences occurred, Reader 2′s estimates were equally younger and older than Reader 1′s estimates (table 2). Moreover, differences were not more prevalent for teeth from the edge. Collectively, these results do not support the hypothesis that increasing AOP on the edge was an artefact of switching tooth readers in 2007.

#### 4.2.2. Hypothesis 2: reduced natural foods

We found no evidence of a wide-scale declining food supply, in either summer or autumn, along the edge of the bear range that could have led to a progressive delay in maturity. It is possible, though, that a single key food had a disproportionate effect on bear reproduction. Severe declines in *Vaccinium* spp. have been linked to dramatic changes in AOP, litter size, litter intervals and/or population declines of bears in western Canada, Sweden and Washington State, USA [63–65]. Acorns or other species of hard mast, where they occur, are typically critical resources for bears [10,66,67]. In Minnesota, bears often migrate well outside their summer home ranges to seek areas with abundant oaks [68]. Our food survey data detected no change in oak abundance along the edge of the bear range, probably because this area is already rich in oak forest. However, we detected an increase in oak abundance in the core of the range, which may partially account for the recent dip in AOP there (figure 4). The US Forest Service Forest Inventory and Analysis data also documented a 25% increase in oak forests statewide from 1990 to 2020 [69,70].

Along the agricultural edge of Minnesota's bear range, bears make heavy use of corn in autumn [54,60]; therefore, if corn production declined, AOP would probably increase. However, both the total area planted and total production of corn steadily increased through the study period, possibly contributing to the rapidly increasing masses of bears. Thus, the perceived increase in AOP on the edge does not appear to be related to reduced foods (table 3).

#### 4.2.3. Hypothesis 3: density-dependent effects

Increased density has led to reduced population growth in brown bears [71], in part owing to males displacing females from the best feeding sites, causing reductions in female masses and reduced reproduction [72]. In brown bears, there is also evidence that increased density of females may suppress the maturation and reproduction of neighbouring young females [73]. However, there is little documentation of density-dependent effects on reproduction in black bears, probably because most populations are hunted and are thus well below their carrying capacity [6,8,74]. In Minnesota, varying hunting pressure over four decades caused bear numbers to initially increase, then decline and then increase again in both the core and the edge (figure 8). Despite a doubling and halving of bear density, AOP was stable in the core and appeared to steadily increase at the edge during both increasing and declining population trajectories (figure 4). Moreover, AOP for edge bears appeared to increase through the 2010s, which, if true, means that reproduction was lowest during the period when the bear population was rapidly surging, and while the number of bears killed by hunters was dramatically increasing [43]. This is obviously a contradiction. Hence, we reject the hypothesis that changes in AOP were driven by density-dependent effects (table 3).

#### 4.2.4. Hypothesis 4: hunter selection for larger juveniles

We considered a scenario in which hunters selectively removed immature bears that were particularly heavy and destined to mature early, leaving slower-growing and hence later-maturing bears in the population. This probably occurred to an extent, particularly on the edge, where 3-year-olds could be nearly as heavy as adults in the core; this situation would also be amplified on the edge, where density of hunters is higher than in the core. Nevertheless, in both areas, bears that survived and went on to become reproductive adults were heavier each succeeding decade (6+ year-olds, figure 7), indicating that these bears and their offspring should have matured young, leading us to reject this hypothesis for the perceived increase in AOP on the edge (table 3).

#### 4.2.5. Hypothesis 5: methodological limits

Taken together, our data on bear body mass, food abundance and population trends strongly suggest that reproduction should not have declined. On the contrary, increases in body mass of young bears, perhaps fuelled by increasing availability of oaks in the core and corn on the edge, should have induced earlier maturation, especially on the edge where 3-year-olds became as heavy as many adults in the core (figure 7). This conundrum prompted us to consider our final hypothesis that the increasing rate of growth of bears on the edge led to a systematic and worsening error in interpreting AOP from cementum annuli.

We hypothesized that better nutrition promoted more reproduction at age 3, but at the same time, the faster growth of these bears made the annuli in the teeth more spread out, so this first litter was harder to detect. This caused an error wherein, for some cases, the second litter at age 5 was identified as the first, resulting in the misperception that AOP was delayed.

This explanation is the only hypothesis that met all predictions (table 3), and is also consistent with our finding that approximately half of the collared bears living along the edge had their first cubs at age 3. However, our tooth-based estimate of AOP indicated that we missed approximately 85% of these. We do not have direct data to show that this error increased with an increasing frequency of AOP at 3 years of age; therefore, this remains an assumption based on increasing masses and increasing corn production.

Coy & Garshelis developed this method for discerning reproduction using teeth from radio-collared bears from the core of the range, where growth was moderate, and few 3-year-olds had cubs [25]. The technique reliably detected known first litters of black bears not only in Minnesota but also in several other states in the USA. Thus, before this study, we had no reason to suspect that there were conditions under which the method might fail.

### 4.3. Use of this method in other states

We contacted 14 states that routinely use teeth from harvested black bears to ascertain reproductive events, but none had examined teeth from local bears with known reproductive histories for reference. However, state agency biologists reported to us that commercial labs reading their teeth sometimes had difficulty discerning reproductive information, particularly in some western and south-eastern states.

Recently, a study in Ontario, Canada, ascertained AOP from a sample of approximately 1000 teeth from black bears harvested in spring or autumn, of which about half showed a cementum pattern indicative of cub production [8]. They observed spatial variation that matched productivity of bear foods, ranging from a mean AOP of 5.1 years in more productive forests to 5.7 years in northern boreal forests. Ontario boreal forest is north of Minnesota's boreal forest, and AOP is accordingly even more delayed than what we observed in the northernmost strata. They also created a bear food index similar to ours and found that food availability was positively related to the probability of a bear producing its first cubs the following year. Their results, like ours, yielded AOP estimates that matched the expectations with regard to nutrition.

Another study in Wisconsin examined more than 19 000 black bear teeth, at least 2-years-old, collected from hunters over 20 years [35]. They reported that a commercial laboratory with considerable experience was able to obtain reproductive data from 25% of this sample of teeth, and among these, approximately half had cementum patterns in which litter production was unclear. Most of the bear range in Wisconsin is geographically continuous with and south of the edge range in Minnesota; therefore, oaks and corn are especially plentiful, and bears grow rapidly, probably contributing to uncertainty in the cementum pattern. They found that approximately 25% of bears produced their first litter at 3 years of age and nearly 80% by age 4, which, although higher than what we found from teeth in the edge in Minnesota, were probably under-estimates as per our direct estimates from radio-collared bears.

These results from neighbouring Ontario (slow-growing bears) and Wisconsin (fast-growing bears) support our hypothesis that the technique works only within limits. Specifically, if the average AOP is greater than or equal to 5 years, it should be readily detected in the teeth of black bears, because by this age, the cementum bands are fairly consistent in width, unlike the earlier years when the tooth is rapidly growing (figure 1). However, in areas where a high proportion of bears produce their first cubs at 4, or especially 3 years of age, the first litter may be less reliably detected. We add, though, that if fast-growing bears produce cubs at an early age, but all the cubs in the litter die (e.g. the Massachusetts example in [49]), this will not confound the reading of AOP in teeth, as only surviving litters (cubs nursed until weaning in autumn) affect the spacing of annuli [25].

## 5. Conclusion

Bear teeth can provide valuable historical data on reproduction in a population. As teeth are routinely collected from successful hunters by state agencies across North America to determine the ages of harvested bears, a large sample of reproductive data is readily available with just an additional few minutes of scrutiny per tooth or nominal additional cost if using a commercial laboratory. Because these teeth are normally archived, data may also be available from previous years, depending on stain retention.

In Minnesota, reproductive data from bear teeth revealed a clear geographic pattern in AOP that made sense in terms of the nutritional landscape of the bear range and indicated long-term stability in AOP in the core of the range. However, this technique produced counter-intuitive results along the edge of the range. Here, we concluded that these results stemmed from an increased rate of growth of young bears (owing to more food), which hindered detection of AOP at 3 years of age.

We offer two key lessons from this work. First, although the tooth-derived data initially misled us, they were valuable in alerting us to the fact that some sort of change was occurring, prompting an exhaustive investigation of multiple hypotheses. Moreover, our concern that AOP might actually be increasing on the edge, signalling a decline in foods that we had not detected from our food surveys, spurred us to expand our field studies in this region, focusing specifically on monitoring young, known-age females through primiparity. What we learned from these radio-collared bears was key to understanding the potential error in reading teeth of fast-growing bears. We recommend that anyone using this technique should be trained using slides of teeth from bears with known AOP. Possibly, knowing this issue, readers can find ways to better detect reproductive events in teeth from places where nutritional resources are consistently very abundant.

The second, more general lesson from this work is about recognizing the limits of indirect measurement techniques such as this one. We had not considered that a technique developed and tested in our state could fail owing to a change as simple as increased nutrition (more corn planted along the edge of the range). In looking for novel techniques to rapidly accumulate large datasets over large spatial areas, we must keep in mind that the conditions for which a technique is developed and tested may not represent the full suite of conditions that may occur, and that its utility should not be assumed to extend beyond those conditions.

## Data Availability

The datasets and code supporting this article are available at [75].
